# Gender Differences in the Incidence of Depression Among Immigrants and Natives in Aragón, Spain

**DOI:** 10.1007/s10903-016-0352-z

**Published:** 2016-02-15

**Authors:** E. M. Esmeyer, R. Magallón-Botaya, A. L. M. Lagro-Janssen

**Affiliations:** 1 Department of Primary and Community Care, Gender and Women’s Health, Radboud University Medical Center, Internal Post 117, P.O. Box 9101, 6500 HB Nijmegen, The Netherlands; 2 Aragon Health Sciences Institute (IACS), Saragossa, Spain

**Keywords:** Depression, Immigrants, Spain, Gender differences, Primary care

## Abstract

Knowledge of depression among immigrants within Spanish primary care is limited. This database study investigates the incidence of depressive disorders among immigrants and natives within primary care in Aragón (Spain). Participants were patients registered in an electronic record register, aged above 20 years diagnosed with depression. Incidence of depression was calculated and compared per continent of origin, gender and age with the Mann-Whitney U test and the Kruskal–Wallis test. The population consisted of 11,088 patients with depression of whom 93.0 % natives and 7.0 % immigrants. Incidence of depression amongst male immigrants was lower than amongst male natives (OR 0.80). Eastern European males showed the lowest incidence 4.1 (3.5–5.3). The gender difference in incidence was larger in immigrants than in natives (OR 3.4 vs. 2.7). Due to male immigrants the incidence of depression within primary care is lower among immigrants. Equal care should be provided to patients of both genders and all origin.

## Introduction

The percentage of foreign-born immigrants in Spain tripled from 4.9 % in 2000 to 14.4 % in 2010, with a total of 6.6 million foreign-born inhabitants in 2010 [1, 2] resulting in the need for the healthcare system to adapt. Immigration exposes immigrants to psycho-social stress during their migration process, which is known to influence mental health and may cause mental disorders [3]. Immigrants are also at higher risk of mood disorders (OR 1.38), as shown in a meta-analysis [4], and region of origin is a significant risk factor in depression [5]. Gender is an important factor relating to depression, as depression is twice as common in women worldwide [6, 7], and there is evidence for a gender gap among immigrants [8–10]. Though the most common mental disorder in Spain is depression, knowledge of depression among immigrants diagnosed by general practioners (GPs) in Spain is limited [11].

Besides immigration and gender, age is an important variable influencing rate of depression. Depressed patients are older and more often female [12]. The rate of major depression doubles after 70–85 years of age, a phenomenon that is assumed to be caused by higher rates of acute and chronic illness, loss of independence, residential housing and social isolation [13–15]. As age influences depression, study of this factor is also required in the immigrant population.

An interesting finding is the lower use of antidepressants among immigrants in Spain [16]. A study focussing on the Spanish region of Aragón also showed that the annual frequency of healthcare visits was lower amongst immigrants [17]. The majority of these previous studies of depression among immigrants, however, are survey studies that do not provide any information about the rate of depression diagnosed by a GP in the primary healthcare system.

In this paper, we set out to investigate whether there are differences in the incidence of depression between male and female immigrants and natives in the primary healthcare system in Aragón, Spain. This region under examination has 1.3 million inhabitants, 12.8 % of whom are immigrants [18]. Unlike other provinces in Spain, virtually all inhabitants of Aragón are registered in its practically free healthcare system [19]. All data for this population are stored in an electronic medical records register and are, therefore, representative of the entire population of Aragón. Our aim is to improve detection and management of depression among immigrants in the Spanish primary healthcare system.

## Methods

### Participants

**Fig. 1:**
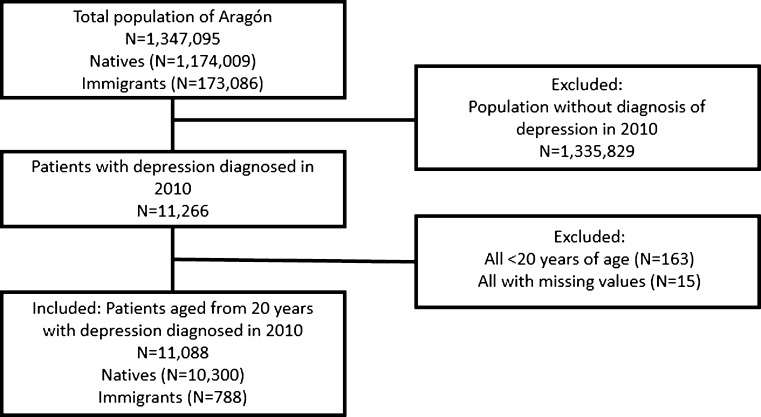
Inclusion of native and immigrant patients with a diagnosis of depression in 2010

Participants were registered patients who were diagnosed with depression in Aragón, Spain. All patients younger than 20 and all patients without information on sex, age, region of origin and with a missing date of diagnosis were excluded from the study. Included patients were labelled as immigrants or natives: all foreign-born patients were defined as immigrants, and all Spanish-born patients were defined as natives. Second-generation immigrants, therefore, were defined as natives. Figure 1 shows the inclusion process of our study.

### Data Collection

In Aragón, all data pertaining to registered patients are coded in an electronic medical records register (OMI©: Computerized Medical Office). Unlike other regions, private healthcare is rare in Aragón, as the Aragonese public system is universal and free, providing access to all inhabitants (1b). We selected all data belonging to patients aged 20 and over who were diagnosed with depression and coded as such (ICPC code P76, [21]) in the year 2010. This code has been defined in the Manual of Mental Disorders (DSM-V) and can only be diagnosed and coded by a GP. Patient anonymity was guaranteed.

### Measures

We identified the following registered characteristics that might influence the incidence of depression: continent of origin (national vs. immigrant; and Africans, Asians, Eastern Europeans, Latin Americans, Northern Americans, and Western Europeans vs. each other), gender (male vs. female) and age group (20–29; 30–39; 40–49; 50–59; 60–69; 70–79; >79 years).

Incidence was defined as the number of new diagnoses of depression per 1000 patients per year. The number of registered depressed patients and the Aragón population as a denominator were used to calculate the incidence. Aragonese population counts, derived from the Spanish National Institute of Statistics [2], were used as a denominator. Rates of incidence per continent of origin, gender and age were calculated.

### Analysis

Non-parametric tests were used to compare the populations of immigrants. The Mann–Whitney U test was used to analyse the difference in the incidence of depression between natives and immigrants. The Kruskal–Wallis test was used to analyse the differences between the populations’ continental origins. All analyses were corrected for confounding by using population characteristics (gender, age and population size). The incidences were expressed as odds ratios (OR) and 95 % confidence intervals (95 % CI). A *p* value of <0.05 was considered statistically significant. This study was approved by the Aragonese Ethic Committee of Clinical Research.

## Results

### Incidence of Depression Among Natives and Immigrants

The population consisted of 11,088 patients with depression, 10,310 (93.0 %) of whom were natives and 778 (7.0 %) of whom were immigrants deriving from 49 different countries. Of all immigrant patients with depression, the largest population (N = 338) was Latin American. The populations of Eastern Europeans (N = 231), Africans (N = 106) and Western Europeans (N = 97) were smaller. The Asian (N = 4) and Northern American (N = 2) populations were too small to analyse.

The median age of the total registered patient population was 57.1 years [56.8–57.4]. The median age was higher among women 57.5 [57.1–57.8] than among men; 56.2 [55.5–56.8]. No age differences were found among immigrants of both sexes. Natives with depression showed a higher median age than immigrants with depression: 58.2 [57.9–58.6] versus 41.7 [40.9–42.6]. The median age of the West Europeans (45.4) was higher than that of the Eastern Europeans (38.8) and Latin-Americans (42.9).

The total population of immigrants showed a significantly lower incidence (OR 0.80) of depression in comparison to natives: 8.2 [7.5–9.11] versus 10.3 [10.2–10.5], with the figures in square brackets denoting the confidence intervals. This is explained by the difference in incidence among male immigrants 3.5 [2.7–4.4] and male natives 6.0 [5.9–6.2]; OR 0.58. Female immigrants and female natives showed no significant difference: 12.8 [11.5–14.3] versus 14.4 [14.2–14.5]; OR 0.89.

### Incidence of Depression per Continent of Origin

**Fig. 2:**
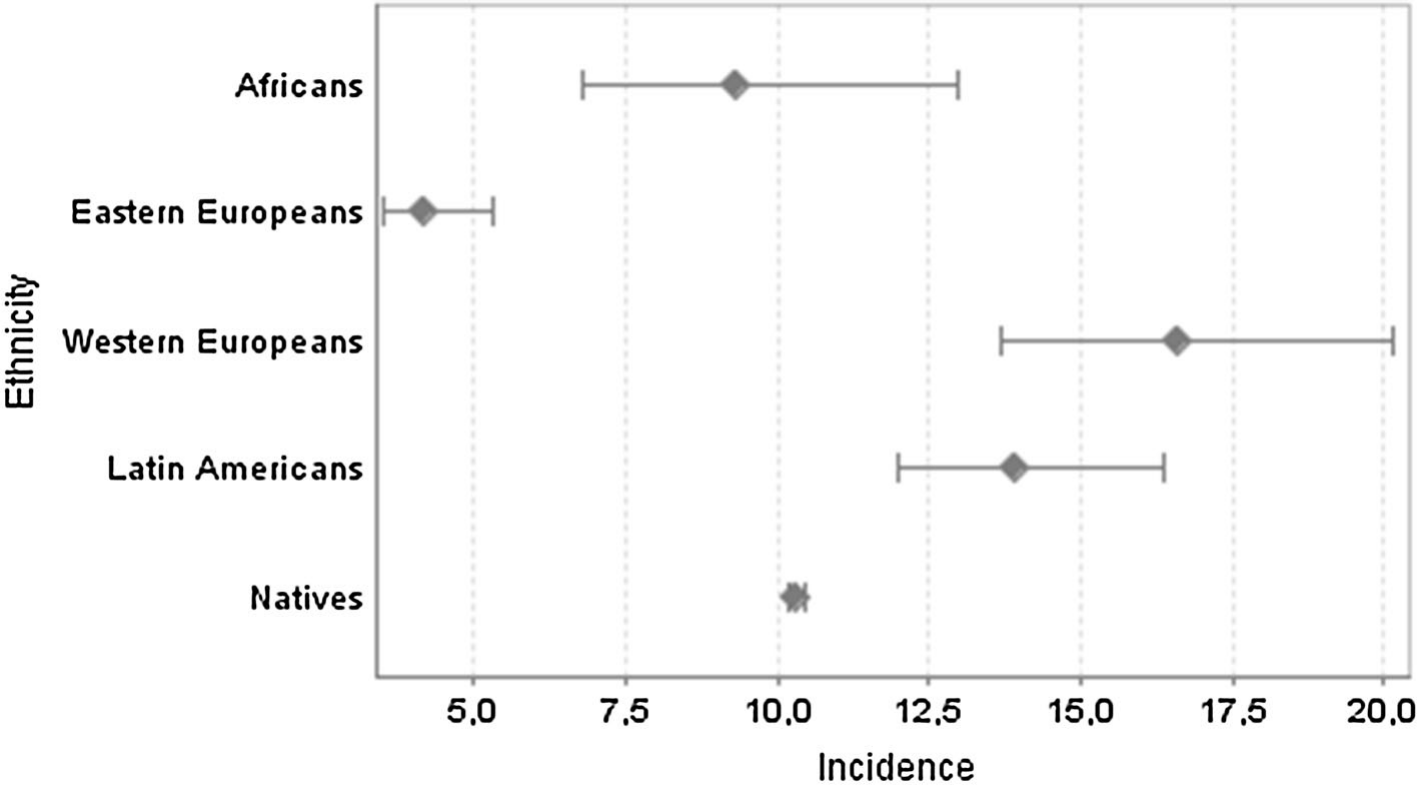
Incidence of depression (95 % CI) by continent of origin

As shown in Fig. 2, the incidence among Western Europeans: 16.6 [13.7–20.1], Latin Americans: 13.9 [12.0–16.4], Africans: 9.3 [6.8–13.0] and natives: 8.2 [8.0–8.4] was higher than the incidence among Eastern Europeans: 4.1 [3.5–5.3]. The incidence among Africans and natives was not significantly different.

### Incidence of Depression per Gender

**Fig. 3:**
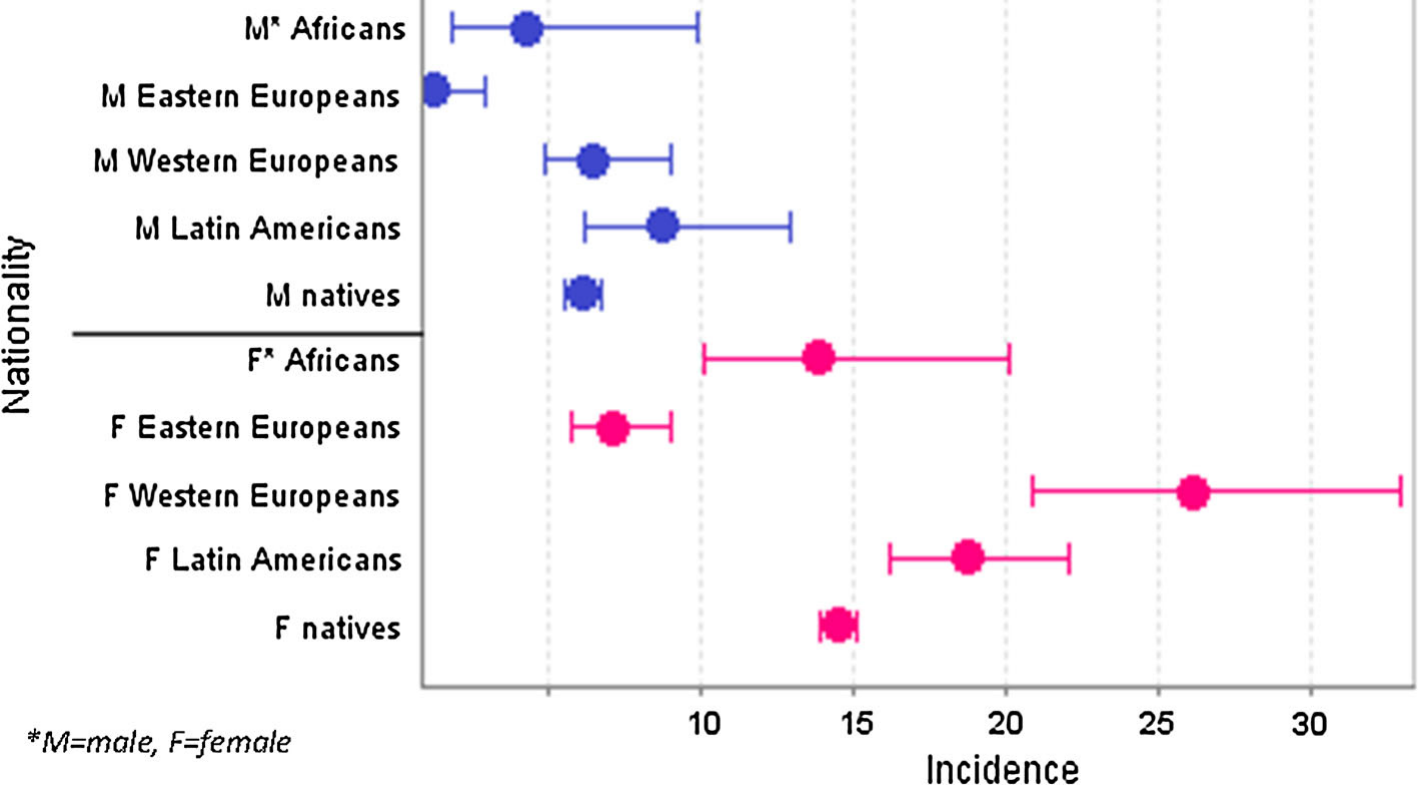
Incidence of depression (95 % CI) per continent of origin analysed by gender

Figure 3 shows the incidence per continent of origin analysed per gender. The incidence among native women was twice as high as among native men: 14.4 [14.2–14.5] versus 6.0 [5.9–6.2] (OR 2.4). The gender difference among female and male immigrants was even larger: 12.8 [11.5–14.3] versus 3.5 [2.7–4.4] (OR 3.7). Women showed higher incidences than men in all age groups. Both sexes displayed a lower incidence among Eastern Europeans in comparison to other origins. Women showed more differences between continents of origin than men. The incidence among female Western Europeans and Latin Americans was higher than that among natives (Fig. 3).

### Incidence of Depression per Age Group

The two youngest age groups of 20–29 and 30–39 years showed a significantly higher incidence of depression among natives when compared to immigrants (OR 0.63 and OR 0.75). The number of immigrants in the categories older than 60 years was too small (N = 33) to analyse. The incidence of depression increases with age in both sexes.

## Discussion

Based on our analysis, we observe a low incidence of depression among immigrants diagnosed by GPs in the Aragonese primary healthcare system. Male immigrants show a lower incidence than male natives, whereas the incidence among female immigrants and natives is comparable. Eastern-Europeans of both sexes show a lower incidence than natives, whereas the Western-Europeans and Latin-Americans show higher incidences than natives. Eastern Europeans, and the males in particular, lower the average incidence among the total population of immigrants. The gender gap is larger among immigrants due to this low incidence among male Eastern Europeans and high incidence among female Western Europeans. This phenomenon might be caused by differences in help-seeking behaviour between male and female immigrants of this origin and gender blindness among GPs.

Besides, OR for depression among immigrants in our study differ clearly from the higher risk found among immigrants in previous population-based surveys [4, 20]. The lower incidence of depression among immigrants, especially males from Eastern Europe, in primary care in our study can be explained by the underdiagnosis of two causes. Firstly, immigrants and natives present their mental problems differently. GPs are possibly more prone to focus on factors such as physical problems, social isolation, poverty and unemployment in the case of immigrants presenting with mental problems than to focus on depression. Secondly, an Aragonese study described a possible lower frequency of healthcare use among immigrants despite the healthcare system being freely accessible [17]. Further study is necessary to improve our understanding of the reasons and the immigrants’ context.

Our study reveals differences in the incidence of depression among populations of different continents of origin. The high incidence among Latin Americans and Western Europeans may be influenced by social isolation, unemployment, lower socio-economic status, discrimination, traumatic life-events, drug use and many other factors. On the other hand, it might be easier for the Spanish-speaking group of Latin Americans to overcome boundaries in visiting a healthcare centre than for immigrants with language problems. The higher frequency of primary healthcare visits in Western Europeans may also be due to their being accustomed to Western healthcare systems. A remarkable finding of our study is the fact that the database contained only four depressive Asian patients out of the total of 4,601 Asian immigrants in Aragón. Asians make a very low use of the Aragonese healthcare system, which may contribute to underestimation of depression within the total group of immigrants [21].

The strength of our study is that it includes the whole population of Aragón, providing a strong basis for an epidemiological study. Its limitations are, firstly, the absence of socio-economic status, marital status, unemployment and traumatising life-events from the medical records register and, therefore, from our study. Secondly, the number of immigrants per continent of origin is too small to be divided in smaller regions of origin. Thirdly, the second generation of immigrants are not considered as immigrants in this study, yet this generation is very small as immigration is a relatively new phenomenon in Spain [1].

## Conclusion

The incidence of depression diagnosed by GPs in the primary healthcare system of Aragón is found to be lower among immigrants than among natives. Male Eastern Europeans show the lowest incidence, which leads to a low incidence among the total group of immigrants. Due to the low incidence among male Eastern Europeans and the high incidence among female Western Europeans, the gender gap is larger in immigrants than in natives. These differences between our findings and those from previous population-based surveys are probably caused by underdiagnosis due to differences in culture, help-seeking behaviour, accessibility of the healthcare system and detection of depression among immigrants by GPs. Both the accessibility of primary healthcare and the detection of depression by GPs need to improve. GPs and other primary healthcare workers should focus on the increasing number of immigrants that makes use of the primary healthcare system. Improvements are necessary in order to provide equal care for patients of both genders and all origins.
